# The role of gastric microecological dysbiosis in gastric carcinogenesis

**DOI:** 10.3389/fmicb.2023.1218395

**Published:** 2023-07-31

**Authors:** Hui Huang, Wei Zhong, Xiaojiao Wang, Ying Yang, Tianmu Wu, Runyang Chen, Yanling Liu, Feng He, Jun Li

**Affiliations:** ^1^Chengdu Medical College, Chengdu, Sichuan, China; ^2^Department of Gastroenterology, The First Affiliated Hospital of Chengdu Medical College, Chengdu, Sichuan, China

**Keywords:** *Helicobacter pylori*, dysbiosis, gastric microbiota, microbial transplantation, gastric cancer

## Abstract

Gastric cancer (GC) is the leading cause of cancer-related death worldwide, and reducing its mortality has become an urgent public health issue. Gastric microecological dysbiosis (including bacteria, fungi, viruses, acid suppressants, antibiotics, and surgery) can lead to gastric immune dysfunction or result in a decrease in dominant bacteria and an increase in the number and virulence of pathogenic microorganisms, which in turn promotes development of GC. This review analyzes the relationship between gastric microecological dysbiosis and GC, elucidates dynamic alterations of the microbiota in Correa’s cascade, and identifies certain specific microorganisms as potential biomarkers of GC to aid in early screening and diagnosis. In addition, this paper presents the potential of gastric microbiota transplantation as a therapeutic target for gastric cancer, providing a new direction for future research in this field.

## 1. Introduction

Gastric cancer (GC) is the fifth most common cancer and the third most common cause of cancer death globally (Smyth et al., 2020). Although treatment strategies for gastric cancer have been continuously updated over centuries, from surgery, chemotherapy, radiotherapy, molecular targeted therapy to immunotherapy, the mortality rate remains high. Addressing the issue of reducing mortality from GC has become an urgent public health concern (Etemadi et al., 2020; Thrift and el-Serag, 2020). GC development involves a multifactorial and dynamic process that results from interaction of various genetic and environmental factors in the host. GC is a cancer type characterized by high heterogeneity. The Cancer Genome Atlas (TCGA) initiated an exhaustive examination that molecularly characterized four subtypes of gastric cancer through genomic analysis, including (i) tumors positive for Epstein–Barr virus (EBV), (ii) tumors with microsatellite instability (MSI), (iii) genomically stable (GS) tumors, and (iv) tumors with chromosomally instability (CIN) (Cancer Genome Atlas Research Network, 2014). GC can be classified into two distinct types, intestinal and diffuse, based on Lauren’s classification (Lauren, 1965). Intestinal GC is well defined by Correa’s cascade alterations that involve a sequence of changes starting from a normal gastric mucosa, erosive gastritis, atrophic gastritis (AG), and intestinal metaplasia (IM), leading to heterogeneous hyperplasia and eventually progressing to *in situ* gastric cancer and invasive carcinoma (Correa et al., 1975).

In recent years, the role of gastric microecological dysbiosis in gastric carcinogenesis has received increasing attention. The gastric microbiota is the most important subset of the gastric microecology. The microbial community in the stomach is predominantly composed of the phyla Firmicutes, Actinobacteria, Bacteroidetes, and Proteobacteria, as well as the genera Lactobacillus, Streptococcus, and Propionibacterium. It has been established that *H. pylori* is a major risk factor for development of GC (Plummer et al., 2016; Alarcón et al., 2017; Yang et al., 2022). In addition to *H. pylori*, other microorganisms (e.g., fungi and viruses) are present in the stomach. The composition of the gastric microbiota can be influenced by various external factors, such as diet, proton pump inhibitors, antibiotics, *Helicobacter pylori* infection, gastric mucosal inflammation, and the mode of delivery at birth. A growing body of research suggests a strong correlation between the gastric microbiota and the occurrence and progression of GC. The gastric microbiota can produce carcinogenic substances, trigger inflammatory reactions, and affect the functionality of immune cells within the immunosuppressive microenvironment, thereby promoting the development of GC. However, the specific composition of the microbiota in gastric tissues remains unclear, and our understanding of how the gastric microbiota changes throughout the different stages of gastric cancer development is still limited. This review provides a detailed analysis of the relationship between gastric microecological dysbiosis and gastric carcinogenesis, along with the potential diagnostic value of the gastric microbiota as a biomarker for GC, which may culminate in the discovery of new diagnostic modalities for gastric cancer. Furthermore, we discuss the potential of gastric/fecal microbiota transplantation as a therapeutic target for gastric cancer, providing a new direction for future research in this area.

## 2. Composition and diversity of gastric microecology

The human gastric microbiota constitutes a distinct microecosystem that participates in maturation and regulation of host metabolism and immunity, as well as inhibition of pathogen colonization. The stomach, with its unique physiological structure characterized by acidic conditions, digestive enzyme secretion, bile reflux, bicarbonate mucus barrier, and gastrointestinal peristalsis, forms a natural screening barrier. Owing to these features, the stomach was once regarded as a sterile organ, and the microbiota was thought to be isolated in the intestine. However, advancements in microbial culture and sequencing techniques have led to the discovery of *Veronococcus*, *Lactobacillus*, *Clostridium*, *Propionibacterium*, *Streptococcus*, and *Staphylococcus* in the human stomach, in addition to *H. pylori* (Marshall et al., 1984; Zilberstein et al., 2007; Delgado et al., 2013). The microbial density of the stomach is approximately 10^1^–10^3^ colony forming units (CFU)/ml, which is substantially lower than that of the intestine (10^10^–10^12^ CFU/ml). The most abundant phyla in the normal gastric microbiota are mainly Bacteroides, Actinomycetes, Firmicutes, Proteobacteria, and Fusobacteria (Bik, 2006; Li et al., 2009). Nevertheless, current techniques far underestimate the diversity of bacteria, as a large proportion of them remain undiscovered by culture (Vartoukian et al., 2010). The microbiota present in the gastric fluid consists mainly of microorganisms from the respiratory tract, oral cavity, and those that retrograde through the intestine into the pylorus (Sanduleanu et al., 2001; Yu et al., 2017). There is significant heterogeneity in the microbiota composition of the gastric mucosa and gastric fluid. Compared to gastric fluid, the gastric mucosa has a greater richness of flora but lower flora diversity (Sung et al., 2016). Many studies have found that the dominant phyla in the normal gastric mucosa are Firmicutes (42%), Bacteroidetes (24%), Proteobacteria (17%), Actinobacteria (7%), and Fusobacteria (6%) (Bik et al., 2006; Delgado et al., 2013; Liu et al., 2019; Pereira-Marques et al., 2019; Ndegwa et al., 2020). In contrast, gastric fluid is mainly composed of the phyla Proteobacteria and Firmicutes (Nardone and Compare, 2015; Sung et al., 2016). These studies revealed a previously unnoticed abundance of the gastric flora and found a heterogeneous community abundance among individuals. Microorganisms in gastric fluid may only reside transiently, without colonizing the gastric mucosa; thus, their diversity is spurious and altered by various factors (Alarcón et al., 2017). Bacterial overgrowth in the stomach has been found in a variety of precancerous conditions, such as hypoacidity and mucosal atrophy. Gastritis caused by chronic *H. pylori* may lead to glandular atrophy and reduced acid production, resulting in a increase in gastric pH. Reduced gastric acid promotes colonization of the gastric mucosa by other bacteria, viruses, and/or fungi (Schulz et al., 2015). These microbes can promote production of nitrite, which in turn leads to accumulation of carcinogenic nitroso compounds, promoting development of gastric cancer.

## 3. Gastric microecological dysbiosis and gastric cancer

**Figure 1:**
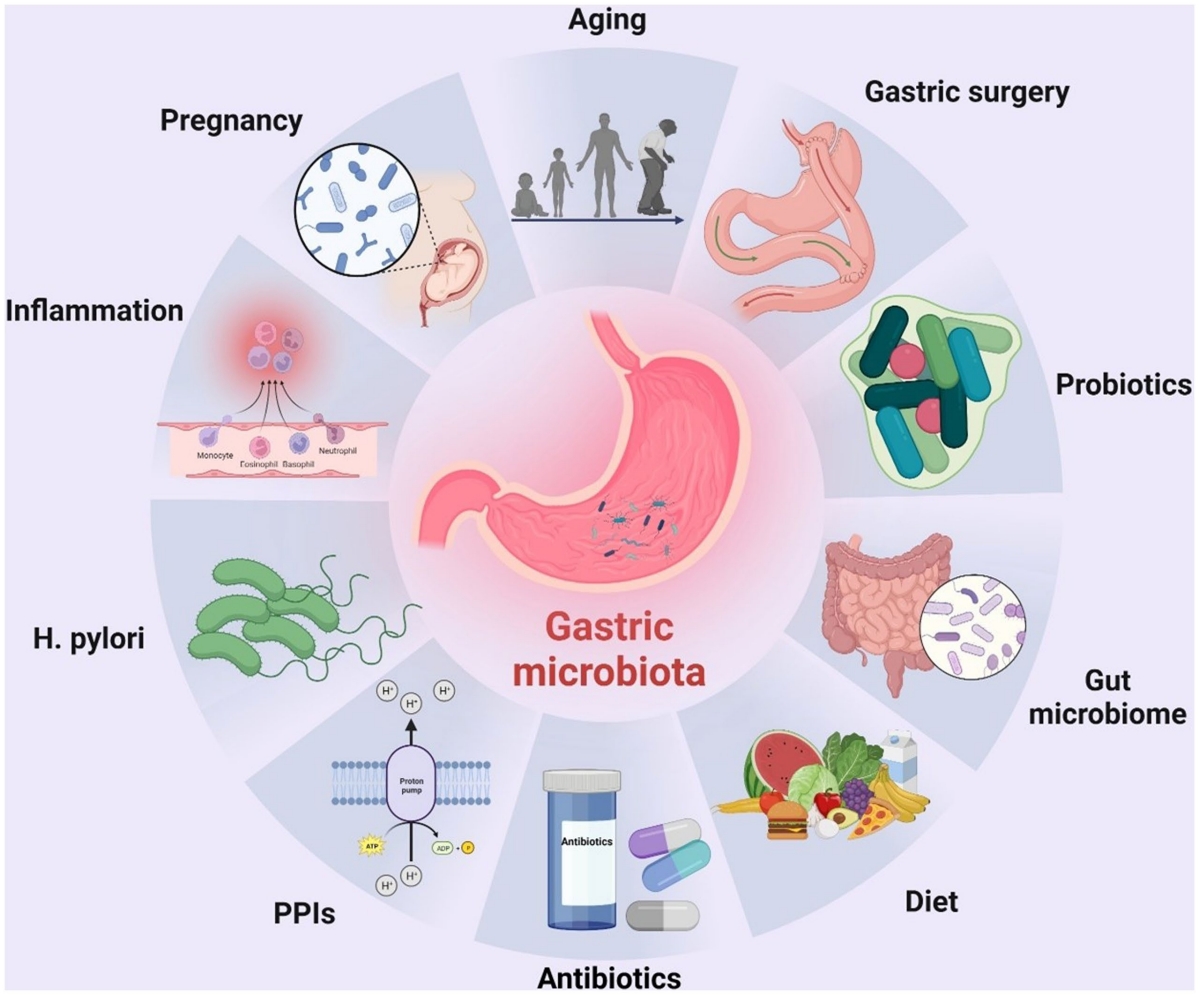
Factors altering the composition of the human gastric microbiota. To date, many factors have been identified that can alter the composition of the human gastric microbiota, including age, surgery, probiotics, gut microbiota, diet, *H. pylori* infection, inflammation of the gastric mucosa, mode of delivery at birth, and use of drugs such as antibiotics and proton pump inhibitors (PPIs). The figure was drawn by BioRender.

In recent years, a growing number of studies have highlighted the role of ecological dysbiosis in cancer development. In a broad sense, ecological dysbiosis refers to changes in the composition and function of the host’s resident microbiota, which can lead to a microecosystem that is conducive to growth and proliferation of cancer cells. The microecosystem comprises a host, a microbiota, and an external environment that can affect the microbiota. The composition of the microbiota is highly dynamic and influenced by several factors, including age, sex, dietary habits, lifestyle, geographic location, *H. pylori* infection, gastric mucosal inflammation, mode of delivery during birth, and use of medications such as antimicrobial agents and proton pump inhibitors (Tsuda et al., 2015; Bokulich et al., 2016; Lloyd-Price et al., 2016; Figure 1). Ecological dysbiosis can manifest in several ways, including loss of beneficial microorganisms, expansion of potentially harmful microorganisms, and reduction in overall microbial diversity (Petersen et al., 2014). These changes create an environment that is favorable for development and progression of cancer, including gastric cancer. In this context, understanding the role of ecological dysbiosis in the pathogenesis of gastric cancer provides important insight into the development of effective prevention and treatment strategies.

### 3.1. Gastric microbiota dysbiosis

#### 3.1.1. Helicobacter pylori

*Helicobacter pylori* has been widely recognized as a crucial risk factor for developing gastric cancer (Touati et al., 2003; Graham, 2015; Hartung et al., 2015; Mégraud et al., 2015), as highlighted by the World Health Organization (WHO), which classifies it as a Group I carcinogen (IARC, 2003; Bouvard et al., 2009). The prevalence of *H. pylori* infection is over 50% worldwide. In China, over 80% of non-cardia gastric cancer cases and over 60% of cardia gastric cancer cases each year can be attributed to *H. pylori* infection (Yang et al., 2021). The bacterium achieves successful colonization in the human gastric mucosal layer through various mechanisms, as follows: (1) using urease and α-carbonic anhydrase to produce ammonia and HCO_3_^2−^ to neutralize gastric acid and raise the pH of local tissues, creating a favorable environment for overproliferation of itself and other bacteria in the stomach (Scott et al., 1998; Celli et al., 2009); (2) enhancing its penetration into the gastric mucosal layer through flagella (Suerbaum, 1995; Martínez et al., 2016); and (3) through bacterial virulence proteins, such as vacuolar cytotoxin (VacA) protein and cytotoxin-associated gene A (CagA) protein. The structure and function of immune cells are regulated in various ways to suppress the body’s immune response to *H. pylori* (Bakhti et al., 2020; Xi et al., 2023). Moreover, *Helicobacter pylori* has the ability to manipulate the physiological functions of gastric epithelial cells by expressing mucins such as Muc1, Muc4, and Muc5b, resulting in loss of cell polarity and release of nutrients and chemokines, including interleukin-8 (IL-8) (Navabi et al., 2013).

The virulence factors CagA and VacA of *H. pylori* are closely associated with GC occurrence, and the carcinogenesis mechanisms among them have been extensively explored. It has been demonstrated that individuals infected with CagA-positive strains are at a higher risk of developing gastric cancer (Blaser et al., 1995; Parsonnet et al., 1997; Huang et al., 2003; Nell et al., 2018). CagA is an oncoprotein that activates multiple signaling pathways, including RAS/ERK, WNT/β-linked protein, JAK/STAT, and PI3K/AKT, and inhibits tumor suppressors, promoting GC (Palrasu et al., 2021). CagA can act as an anti-apoptotic protein that inhibits pro-apoptotic factors such as SIVA1, BIM, and BAD; it also regulates autophagy and induces inflammation (Vallejo-Flores et al., 2015; Palrasu et al., 2020). Humans infected with CagA-positive *H. pylori* strains are known to exhibit an intense inflammatory response and severe damage to gastric tissues (Yamaoka et al., 1997; Figura et al., 1998). The interaction of CagA with Lactobacillus enhances maturation of human monocyte-derived dendritic cells (DCs) and induction of inflammatory mediators other than *H. pylori* (Castaño-Rodríguez et al., 2017). This suggests that bacteria capable of producing lactic acid may increase the inflammatory response induced by *H. pylori*, thus promoting gastric carcinogenesis. CagA was also found to promote the Ye s-Associated-Protein (YAP) signaling pathway, thereby promoting the epithelial-mesenchymal transition (EMT) and gastric carcinogenesis (Li et al., 2018). The EMT causes epithelial cells to lose their characteristic cell–cell contact and become more migratory and invasive by a process that may contribute to the ability of *H. pylori* to penetrate deeper into the gastric mucosa. VacA, another virulence factor of *H. pylori*, is associated with a variety of functions including disruption of the gastric mucosal barrier, interference with antigen presentation pathways, and downregulation of autophagy and phagocytosis, which contribute to bacterial inhibition of immune cells and establishment of persistent infection (Atherton et al., 1995; Boncristiano et al., 2003; Sundrud et al., 2004). The ability of VacA to downregulate autophagy and lysosomal degradation contributes to accumulation of CagA in gastric epithelial cells (Abdullah et al., 2019).

Moreover, *H. pylori* significantly inhibits colonization of the stomach by other bacteria, resulting in lower gastric microbiota diversity (Gao et al., 2018). In female BALB/c mice without specific pathogens, colonization by *H. pylori* leads to an increase in the gastric microbiota of *Clostridia*, *Bacteroides*/*Prevotella* spp., *Eubacterium* spp., *Ruminococcus* spp., *Streptococci*, and *Escherichia coli*, with decreases in lactobacilli (Aebischer et al., 2006). An early study used 16S rRNA microarrays to analyze gastric biopsies from subjects infected or not with *H. pylori* (Maldonado-Contreras et al., 2011). This study found that *H. pylori*-positive patients had a higher abundance of Proteobacteria, Firmicutes, Bacteroides and Actinobacteria in the stomach and a lower abundance of Actinobacteria, Bacteroidetes, and Firmicutes. All the above studies reveal a significant effect of *H. pylori* colonization on the gastric microbial community. However, other studies have clearly shown that the gastric microbiota is not altered by chronic *H. pylori* infection (Tan et al., 2007; Khosravi et al., 2014; Coker et al., 2018). Thus, the relationship between *H. pylori* and other flora remains controversial (Table 1).

**Table 1:** Possible roles of *Helicobacter pylori* and *non-Helicobacter pylori* bacteria in the development of gastric cancer.

Microbiota	Effects on the development of GC	Mechanisms	Reference
*H. pylori*	Interfering with repair gene expression	The mechanism of mismatch repair (MMR) is responsible for preserving genomic stability by rectifying mistakes that occur during the replication of DNA. In patients with gastric cancer who are microsatellite instability (MSI)-positive, it has been observed that the activity of *H. pylori* infection is more pronounced compared to those who are MSI-negative. This suggests that *H. pylori* may play a role in influencing the DNA MMR system during gastric carcinogenesis.	Leung et al. (2000)
	Promoting inflammatory response	The virulence factor CagA, which is produced by *H. pylori*, activates an inflammatory, NF-κB-dependent signaling pathway. This leads to the recruitment of inflammatory cells, as well as reactive oxygen species-induced injuries and wound healing responses. These mechanisms play a role in promoting gastric carcinogenesis.	Brandt et al. (2005) and Amieva and Peek (2016)
	Regulating the function of immune cells in TME	The infection caused by *H. pylori* triggers the upregulation of fibroblast activating protein (FAP) and fibroblast surface protein (FSP) mRNA, as well as elevated levels of pro-inflammatory factors such as IL-6, IL-8, COX-2, and SDF-1. Overexpression of FAP hinders the regulation of fibroblast growth, obstructs tissue repair, and promotes the progression of epithelial-mesenchymal transition (EMT) and the development of gastric cancer.	Krzysiek-Maczka et al. (2018) and Baj et al. (2020)
	Regulating production of metabolites for GC development	*Helicobacter pylori*-derived CagA has been shown to induce the expression of spermine oxidase (SMOX), which leads to the production of hydrogen peroxide (H_2_O_2_). The increased H_2_O_2_ levels may in turn lead to ROS accumulation via mitochondrial membrane depolarization and activation of cysteine-mediated apoptosis. This pathway may contribute to the development of gastric cancer.	Chaturvedi et al. (2004)
*Non-H. pylori*			
*Propionibacterium acnes*	Promoting inflammatory response	*Propionibacterium acnes* is potentially implicated as the pathogenic agent responsible for lymphocytic gastritis. It is surmised that the condition is dependent upon the natural killer factor group 2 member D (NKG2D) system, along with the pro-inflammatory cytokine IL-15. The activation of these signaling pathways is postulated to potentially promote the progression of gastric cancer.	Montalban-Arques et al. (2016) and Gunathilake et al. (2019)
*Lactobacillus murinus*	Promoting inflammatory response	In a transgenic INS-GAS mouse model, the gastric lesions instigated by the overgrowth of *Lactobacillus murinus* ASF361 were found to be correlated with the robust expression of molecules associated with gastric inflammation and cancer, including TNF-α, Ptger4, and TGF-β.	Lertpiriyapong et al. (2014)
Stenotrophomonas, Acinetobacter, Haemophilus	Regulating the function of immune cells in TME	The presence of Stenotrophomonas, Acinetobacter, and Haemophilus in GC tissues showed a positive correlation with BDCA2+ pDC. The pDCs were responsible for the generation of CD4+ CD25+ Foxp3+ Treg cells, leading to functional incompetence and immunosuppression, thereby facilitating immune evasion by tumor cells.	Ling et al. (2019)
*F. nucleatum*	Regulating the function of immune cells in TME	Cancerous tissue is targeted by *F. nucleatum* via an interaction between the Fusobacterium lectin Fap2 and the tumor-specific surface Gal-Gal NAc. This attachment elicits the expression of MUC2 and TNF-α in colon cancer cells. *F. nucleatum* is one of the strains that is enriched in the gastric cancer microbiota, and considering its significance in colorectal cancer, *F. nucleatum* could also play a pivotal role in the development of gastric cancer.	Coppenhagen-Glazer et al. (2015) and Hsieh et al. (2018)
Clostridium	Regulating production of metabolites for GC development	*Clostridium perfringens* can impact bile acid metabolism, which has been implicated in the pathogenesis of gastric cancer. Additionally, it has been discovered that this pathogen plays a role in gastric carcinogenesis through the upregulation of histidine decarboxylase (HDC).	Ridlon and Bajaj (2015) and Lee et al. (2020)
Lactic acid bacteria (LAB)	Regulating production of metabolites for GC development	LAB has been demonstrated to elicit the secretion of extrinsic lactic acid, ROS, and N-nitroso compounds, which can lead to DNA damage and hasten the onset of carcinogenesis. Furthermore, LAB has been observed to augment the expression of proto-oncogenes, stimulate angiogenesis, impede the apoptotic process, and intensify EMT, immune tolerance, and the colonization of other carcinogenic pathogens. Taken together, these events have the potential to propel the progression of gastric cancer.	Vinasco et al. (2019) and Whiteside et al. (2021)

GC, gastric cancer; MMR, mismatch repair; MSI, microsatellite instability; CagA, cytotoxin-associated gene A;TME, tumor microenvironment; FAP, fibroblast activating protein; FSP, fibroblast surface protein; COX-2, cyclooxygenase-2; SDF-1, stromal cell-derived factor-1; EMT, epithelial-mesenchymal transition; SMOX, spermine oxidase; ROS, reactive oxygen species; NKG2D, natural killer factor group 2 member D; INS-GAS, insulin-gastrin; Gal-Gal NAc, *N*-acetylgalactosamine; TNF-α, tumor necrosis factor-α; Ptger4, prostaglandin E receptor 4; TGF-β, transforming growth factor-β; BDCA2, blood dendritic cell antigen 2; pDC, plasmacytoid dendritic cell; Foxp3, forkhead box P3; MUC2, Mucin-2; HDC, histidine decarboxylase.

#### 3.1.2. The bacteria beyond *Helicobacter pylori*

Recent research has suggested that the presence of microbes other than Hp may also contribute to the occurrence of GC. Insulin-gastrin-secreting (INS-GAS) mice are genetically susceptible to GC and are therefore commonly used in developing mouse gastric cancer models. Tumorigenesis is relatively delayed in mice infected with *H. pylori* alone compared to INS-GAS mice infected with *H. pylori* and other gastric microbiota (Lee et al., 2008). Furthermore, *H. pylori*-infected germ-free (GF) INS-GAS mice exhibit less severe gastric mucosal lesions and slower tumor progression than *H. pylori*-infected specific pathogen-free (SPF) INS-GAS mice (Lofgren et al., 2011). These findings suggest that GC may be promoted by microorganisms in addition to *H. pylori*, with other gastric microbial communities also playing a potential role in the carcinogenesis and progression of GC in mice.

In another study, the stomachs of *H. pylori*-infected INS-GAS mice were colonized with different types of intestinal microorganisms, including restricted altered Schaedler flora (rASF; consisting of *Clostridum* species ASF356, *Bacteroides* species ASF519 and *Lactobacillus murinus* ASF361) and pathogen-free (complex IF) mice (Lertpiriyapong et al., 2014). The results showed that the mice colonized by either rASF Hp or IF Hp exhibited severe pathology. The IF Hp-colonized mice showed the strongest inflammatory response, with 40% developing invasive gastrointestinal intraepithelial neoplasia (GIN). The same phenomenon was observed in 23% of the rASF Hp mice. Moreover, it was found that ASF and *H. pylori* coinfection leads to gastric mucosal changes in mice; gastritis in mice was accompanied by reduced colonization of *Clostridium perfringens* ASF356 and *Bacillus mimicus* ASF519 and overgrowth of *Lactobacillus murinus* ASF361.

A recent study conducted by Kwon et al. (2022) inoculated gastric tissue and gastric fluid from patients with chronic superficial gastritis (CSG), intestinal metaplasia (IM) or gastric cancer (GC) into GF C57BL/6 mice. The gastric microbiota of the mice was analyzed by amplicon sequencing and immunohistochemical analysis of the histopathological features of the stomach of the mice. The results revealed that when microbiomes from IM or GC patients were transplanted into the stomachs of GF mice, precancerous features were induced, including increased inflammation, decreased mural cells, and increased cell proliferation. In addition, long-term observations of mice inoculated with the microbiota from IM or GC patients led to a relatively high incidence of features of gastric dysplasia in mice. From the above evidence, it appears that commensal microorganisms in the stomach other than *H. pylori* are associated with development of GC.

#### 3.1.3. Fungi

Fungal species can be detected in the gastrointestinal tract of approximately 70% of healthy adults. The number of fungi in the human stomach is 0–10^2^ cfu/ml, and *Candida* is the most common species (Schulze and Sonnenborn, 2009; Zwolinska-Wcisło et al., 2009). A study examined gastric fluid from 25 patients undergoing clinical indications using PCR amplification of the internal transcribed spacer region, with *Candida* and *Phaalemonium* found to be the only two genera present in all gastric fluid samples (von Rosenvinge et al., 2013). *Candida albicans* can survive under acidic conditions of pH 1.4 and above, and specific genotypes such as DST1593 may exacerbate the severity of gastric mucosal lesions (Gong et al., 2012). *Candida albicans* was detected in 54.2% of gastric ulcer cases and in 10.3% of chronic gastritis cases in a fungal analysis of biopsies from 293 patients with clinical manifestations of dyspepsia or ulcer disease (Karczewska et al., 2009). Gastric fungal infections and colonization are common in patients with GC, and chronic ingestion of exogenous mycotoxins from spoiled foods is a common cause of GC due to fungi. Candida infection is present in 20% of patients with gastric cancer (Scott and Jenkins, 1982). A study performed ITS rDNA genetic analysis of GC-associated fungal composition in cancerous lesions and paracancerous and noncancerous tissues from GC patients, reporting identification of 17 fungal species with significant differences between the two groups at the family level. In the GC group, Pseudeurotiaceae, Trimorphomycetaceae, Chaetomiaceae, and Aspergillaceae were significantly decreased and Saccharomycetales_fam_Incertae_sedis and Pleosporaceae increased compared to the control group. In addition, at the genus level, there were 15 different fungi between the two groups, and two fungal genera were enriched in the GC group, including Candida and Alternaria, whereas Saitozyma and Thermomyces were depleted (Zhong et al., 2021).

#### 3.1.4. Viruses

In addition to the microorganisms mentioned above, EBV can also cause an imbalance in gastric microecology and promote gastric cancer (Martínez-López et al., 2014). More than 90% of adults are infected with EBV, and EBV-associated gastric cancer (EBVaGC) accounts for 5–20% of all gastric cancer cases worldwide (Takada, 2000; Iizasa et al., 2012). Recent studies have described a synergistic role between *Helicobacter pylori* and EBV in gastric carcinogenesis. Individuals coinfected with *H. pylori* and EBV exhibit more severe inflammatory lesions than those infected with *H. pylori* alone (Cárdenas-Mondragón et al., 2013). In addition, some studies have shown that colonization by *H. pylori* induces reactivation of latent EBV in gastric epithelial cells (Minoura-Etoh et al., 2006; Shukla et al., 2012). Proliferation of lymphocytes after EBV infection and their ability to interact with immune effects may be directly influenced by the presence of bacterial or other microbial components at the site of infection (Iskra et al., 2010). These reports suggest that infections caused by dysbiosis may activate latent EBV, thereby increasing risk of developing cancers associated with EBV infection (Matsusaka et al., 2014; Nishikawa et al., 2014; Rickinson, 2014). Although EBV has been suspected of causing several upper gastrointestinal diseases, most studies to date have been case reports, and large-scale studies to support a causal relationship between EBV and these diseases are lacking (Hisamatsu et al., 2010; Owens et al., 2011). In addition to EBV, various other viruses have been found to contribute to tumorigenic changes in the stomach. These viruses include John Cunningham virus (JCV) (Murai et al., 2007), human cytomegalovirus (HCMV) (Jin et al., 2014; Fattahi et al., 2018; Kosari-Monfared et al., 2019), hepatitis B (HBV) or C (HCV) viruses (Song et al., 2019; Cui et al., 2020; Huang et al., 2020), human immunodeficiency virus (HIV) or human T-cell lymphophilic virus (HTLV) (Matsumoto et al., 2018; Kang et al., 2019; Schierhout et al., 2020), and papillomavirus (HPV) (Zeng et al., 2016; Bozdayi et al., 2019).

### 3.2. Other clinical conditions

#### 3.2.1. Acid inhibitors

The presence of hydrochloric acid within the gastric fluid serves as a barrier to many microorganisms, protecting the stomach against potential infections (Yoshiyama and Nakazawa, 2000; Beasley et al., 2015). Over the past decade, acid-suppressing drugs such as proton pump inhibitors (PPIs) and H2 receptor antagonists (H2RAs) have been frequently used to treat gastrointestinal disorders, which have led to significant changes in the microbial diversity of the stomach (Fisher and Fisher, 2017). This shift can trigger hypochlorhydria, a condition that reduces microbial diversity, fosters proliferation of genotoxic microorganisms, and heightens activity of bacterial nitrate/nitrite reductases, ultimately leading to conversion of nitrite and other nitrogenous compounds in gastric fluid to cancer-associated *N*-nitroso compounds (Correa, 1992; Ahn et al., 2013; Ferreira et al., 2018). Some bacteria have been isolated from the stomachs of patients with hypochlorhydria, including *Lactobacillus*, *Streptococcus*, *Pseudomonas*, *Xanthomonas*, *Proteus*, *Klebsiella*, *Neisseria*, *E. coli*, and *Campylobacter jejuni* (Williams and McColl, 2006).

Acid-suppressing drugs have also been shown to impact progression of *H. pylori*, thereby promoting gastric carcinogenesis. In the case of reduced gastric acid secretion (caused by acid-suppressive drugs or chronic atrophy), *H. pylori* leads to a shift from sinusoidal to gastric body-dominated gastritis (Malfertheiner et al., 2017), reducing the lining cells and potentially enhancing RONS-mediated gastric mucosal damage (Suzuki et al., 1992), all of which are associated with increased risk of gastric cancer (Moayyedi et al., 2000; Sanduleanu et al., 2001). Acid-suppressing drugs have been shown to have negative effects on gastric function and host defense mechanisms, ultimately leading to delayed gastric emptying, decreased gastric mucus viscosity, increased gastric pH, increased bacterial load, and increased bacterial translocation (Wandall, 1992; Scarpignato et al., 2016). Several studies have documented changes in the gastric microbiota of patients treated with PPIs versus those not treated, with reduced bacterial clearance noted in the former (Tsuda et al., 2015; Paroni Sterbini et al., 2016). As the duration of PPI treatment increases, there is a gradual rise in the number of culturable bacteria in the gastric lumen and mucosa (Sanduleanu et al., 2001; del Piano et al., 2012), with maintenance treatment for more than 1 year resulting in a 10^6^-fold increase in CFU counts (del Piano et al., 2012). Other studies have reported an increased risk of gastric cancer in long-term PPI users, with or without *H. pylori* eradication, up to 2.4 times higher than in nonusers (Ahn et al., 2013; Brusselaers et al., 2017; Cheung et al., 2018).

Interestingly, two recent meta-analyses suggest that current evidence does not support that maintenance PPI can cause or accelerate development or progression of gastric precancerous lesions such as gastric atrophic changes, intestinal chemosis, intestinal chromophobic (ECL) cell hyperplasia and heterogeneous hyperplasia (Eslami and Nasseri-Moghaddam, 2013; Song et al., 2018). Therefore, further prospective studies are needed to elucidate how maintenance antacids lead to alterations in the microbiota and whether such alterations ultimately increase risk of GC.

#### 3.2.2. Antibiotics

In general, application of antibiotics fundamentally alters the normal microbial community of the body, and gastric microecology is no exception. For example, treatment with cefoperazone sodium/sulbactam sodium disrupts gastric microecology, resulting in overproliferation of enterococci and a marked decrease in lactobacilli. A meticulous investigation of bacterial and fungal microbiota conducted in 25 dyspeptic patients demonstrated that antibiotics lead to reduced bacterial colonization while having a negligible effect on fungal diversity (Eslami and Nasseri-Moghaddam, 2013). Nevertheless, exposure to penicillin causes yeast overgrowth in gastric epithelial cells in mice (Nardone and Compare, 2015), whereas germ-free mice exposed to cefoperazone develop gastritis due to an increase in *Candida albicans* (Mason et al., 2012). A 15-year long-term study comprising 3,365 individuals diagnosed with *H. pylori* infection revealed that short-term treatment with amoxicillin and omeprazole reduces the incidence of gastric cancer by 39% overall (OR = 0.61, 95% CI = 0.38–0.96, *p* = 0.032) (Ma et al., 2012). Interestingly, eradication of *H. pylori* failed in more than half of the individuals treated with antibiotics at the 15-year follow-up, suggesting that antibiotic therapy may curb development of gastric cancer by instigating alterations in the non-*H. pylori* microbiota. It is worth noting that the eradication rate of *H. pylori* during the follow-up period was only 47%, highlighting that antibiotic intervention can possibly mitigate progression of gastric cancer by inducing modifications in the non-*H. pylori* microbiota.

#### 3.2.3. Gastric surgery

Alterations in the anatomy of the gastric environment can also affect its microbiota composition. Roux-en-Y gastric bypass surgery, a common treatment for morbid obesity, involves division of the stomach into two parts, a small proximal pouch and a larger bypass chamber. Postoperative comparison of pH, microbial counts, and mucosal cytokine levels between these two gastric pouches revealed a neutral pH in the proximal gastric capsule (pH 7.0 ± 0.2), with a significant increase in the number of aerobic and anaerobic bacteria, whereas the pH of the anastomotic capsule decreased to 3.3 ± 2.2, with a clear decrease in the total number of bacteria (Faintuch et al., 2007; Ishida et al., 2007). Although no differences in microbiota composition were detected between the two pouches, this is likely due to the limited number of isolates obtained. Residual gastric cancer frequently occurs after distal gastrectomy of benign lesions, with an incidence rate of 1–7% among all gastric cancers (Lagergren et al., 2012). EBV infection has been associated with residual gastric cancer, and atrophic changes in residual gastritis often accompany EBV-positive residual gastric cancer. Moreover, Billroth-II anastomosis (Nishikawa et al., 2002; Kaneda et al., 2012) can significantly alter the gastric microbiota by reducing levels of nitrate and nitrite reductase and expression of nitrosation-related genes, which greatly increases risk of gastric cancer. The diversity of the microbiota in the stomach also becomes richer after subtotal gastrectomy (Clyne and May, 2019). Preoperatively, *Ralstonia* and *Helicobacter* were the two dominant genera identified in gastric cancer, and *Streptococcus* and *Prevotella* were the two most abundant genera in the gastric mucosal microbiome after gastric lesion resection. Overall, these findings suggest that gastric surgery is a key factor in determining the composition of the gastric microbiota.

## 4. Gastric microbial dysbiosis in Correa’s cascade

**Figure 2:**
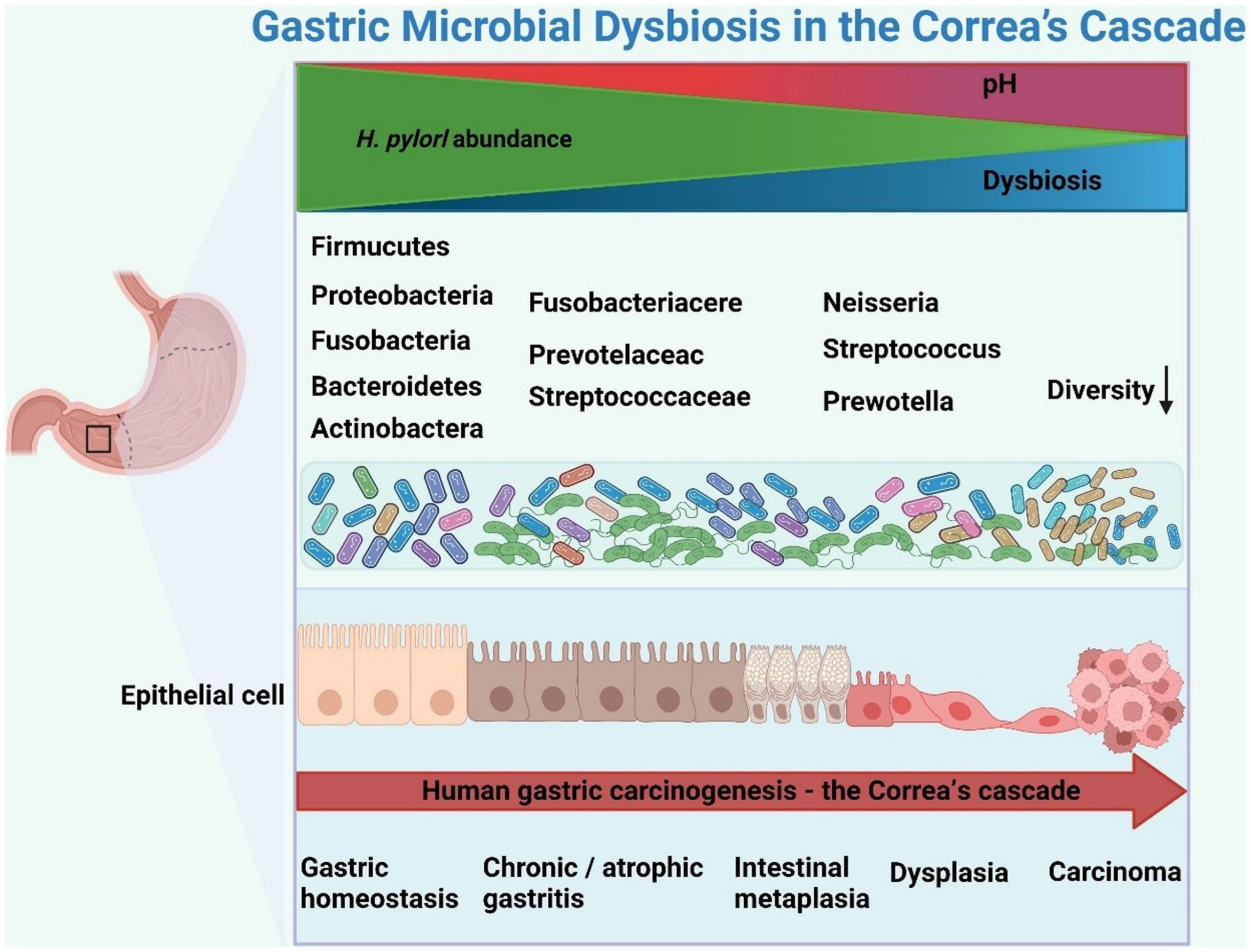
Schematic representation of gastric microbial dysbiosis in Correa’s cascade. *Helicobacter pylori* infection triggers an inflammatory cascade, altering the gastric microbiota and promoting gastric cancer through dysbiosis and inflammation. The figure was drawn by BioRender.

Correa’s cascade refers to a sequence of pathological changes that occur in the gastric mucosa over time, including chronic atrophic gastritis (CAG), intestinal metaplasia (IM), and dysplasia, eventually progressing to invasive carcinoma (Figure 2). The network of characteristics, functions and interactions of the gastric microbiota varies according to the stage of Correa’s cascade (Ferreira et al., 2018; Zhang et al., 2021; Table 2). The current challenge in studying the gastric microbiota is the lack of standardized methods for sample collection, processing and analysis. Different sequencing platforms, sample types (e.g., biopsy vs. aspirate), and analytical methods (e.g., OTU clustering vs. exact sequence variants) can all impact the results obtained. Additionally, differences in the regions of the 16S rRNA gene sequenced also affect the accuracy of the results. This lack of standardization makes it difficult to compare results across different studies, which can lead to conflicting conclusions.

**Table 2:** Dysbiosis of the gastric microbiota in gastric carcinogenesis.

Year	Method	Country	Sample size	Key results	Reference
2009	16S rRNA gene seq	Sweden	Controls (*n* = 5); GC (*n* = 10).	*Streptococcus*, *Lactobacillus*, *Veillonella*, and *Prevotella* dominated GC microbiota, while the abundance of *H. pylori* was relatively low.	Dicksved et al. (2009)
2014	16S rRNA gene microarray	Mexico	NAG (*n* = 5); IM (*n* = 5); GC (*n* = 5).	Bacterial diversity steadily reduced from NAG to IM to GC. A significant difference in microbiota was observed between NAG and GC.	Aviles-Jimenez et al. (2014)
2014	16S rRNA gene seq	South Korea	CG (*n* = 10); IM (*n* = 10); GC (*n* = 10).	The relative abundance of Helicobacteraceae in GC was significantly lower compared to CG and IM, while the relative abundance of Streptococcaceae was obviously increased.	Eun et al. (2014)
2016	16S rRNA gene	China	CG (*n* = 212); GC (*n* = 103).	Five genera of bacteria with potential cancer-promoting activities were enriched in gastric cancer, including *Lactobacillus*, *Escherichia*, *Shigella*, Nitrospirae, *Burkholderia fungorum*, and Lachnospiraceae.	Wang et al. (2016)
2017	16S rRNA gene profiling	Portugal	CG (*n* = 81); GC (*n* = 54).	*Streptococcus*, *Prevotella*, and *Neisseria* enrichment in chronic gastritis was demonstrated. *Achromobacter*, *Citrobacter*, *Phyllobacterium*, *Clostridium*, *Rhodococcus*, and *Lactobacillus* showed a significant increase in abundance in GC.	Ferreira et al. (2018)
2018	16S rRNA gene	China	SG (*n* = 21); AG (*n* = 23); IM (*n* = 17); GC (*n* = 20).	*Peptostreptococcus stomatis*, *Streptococcus anginosus, Parvimonas micra, Slackia exigua*, and *Dialister pneumosintes* were significantly increased in GC. Vogesella, Candidatus Portiera, Comamonadaceae, and Acinetobacter were depleted.	Coker et al. (2018)
2019	16S rRNA gene	China	Normal (*n* = 230); peritumoral tissues (*n* = 247); tumoral tissues (*n* = 229).	*Helicobacter*, *Halomonas*, and *Shewanella*, were enriched in the peritumoral microhabitat, while *Streptococcus*, *Selenomonas*, *Fusobacterium*, *Propionibacterium*, and *Corynebacterium* were enriched in the tumoral microhabitat.	Liu et al. (2019)
2019	16S rRNA gene	South Korea	Controls (*n* = 288); GC (*n* = 268).	The relative abundance of *Helicobacter*, *Propionibacterium*, and *Prevotella* in the GC group was higher than that in the control group, while the relative abundance of Lactococcus in the control group was higher than that in the GC group.	Gunathilake et al. (2019)
2019	16S rRNA gene	China	Paired tumor and nontumor samples from GCA patients (*n* = 36).	GCA tumor tissues had increased relative abundances of *Firmicutes*, *Bacteroidetes*, *Fusobacteria*, and *Actinobacteria* but decreased relative abundances of *Proteobacteria* compared to nontumor tissues.	Shao et al. (2019)
2020	16S rRNA gene seq	China	Normal (*n* = 20); NAG (*n* = 20); AG (*n* = 40); IM (*n* = 40); GC (*n* = 48).	*Parvimonas*, *Streptococcus*, *Peptostreptococcus*, *Fusobacterium*, *Glutamicibacter*, and *Escherichia* were enriched in the GC group.	Gantuya et al. (2020)
2020	16S rRNA gene seq	Italy	Paired tumor and nontumor samples from SRCC (*n* = 10) and ADC (*n* = 10) patients.	SRCCs were significantly enriched in the phyla *Fusobacteria*, *Bacteroidetes*, and *Patescibacteria*, whereas in the ADC type, *Proteobacteria* and *Acidobacteria* phyla were found.	Ravegnini et al. (2020)
2021	ITS rDNA seq	China	Paired tumor and nontumor samples from GC patients (*n* = 45).	With the significant increase of *C. albicans* in GC, the abundance of *Fusicolla acetilerea, Arcopilus aureus*, and *Fusicolla aquaeductuum* were increased, while *Candida glabrata, Aspergillus montevidensis, Saitozyma podzolica*, and *Penicillium arenicola* were obviously decreased.	Zhong et al. (2021)
2021	16S rRNA gene	China	CG (*n* = 25); GC (*n* = 34)	A significant abundance of *Gammaproteobacteria* with the specific order *Peudomonodales* were found only in *H. pylori*-negative gastric cancer patients, while an abundance of the phylum Bacteroidetes and its specific order of Bacteroidales were observed in *H. pylori*-positive gastric cancer patients in addition to Proteobacteria taxa and its specific order Campylobacterales.	Deng et al. (2021)
2022	ITS rDNA seq	China	Paired tumor and nontumor samples from GC (*n* = 61) patients.	Solicoccozyma was significantly enriched in the tumor group, while the abundance of Pezizomycetes, Sordariales, Chaetomiaceae, and Rozellomycota was lower than in the normal group.	Zhang et al. (2022)
2022	16S rRNA gene sequencing	China	Paired GM and GF samples from SG (*n* = 61), IM (*n* = 55), and GC (*n* = 64) patients.	The abundances of some NOC-producing genera, such as *Veillonella*, *Haemophilus*, and *Peptostreptococcus*, in GM approached that of GF, with no significant difference in patients with GC, while their abundances were strikingly higher in GF than GM in patients with SG.	He et al. (2022)

AG, atrophic gastritis; ADC, adenocarcinoma; CG, chronic gastritis; GC, gastric cancer; GCA, gastric cardia adenocarcinoma; GM, gastric mucosa; GF, gastric fluid; IM, intestinal metaplasia; ITS, internal transcribed spacer; NAG, nonatrophic gastritis; SG, superficial gastritis; SRCC, signet-ring cell carcinoma.

### 4.1. Precancerous state

The precancerous state of chronic atrophic gastritis (CAG) and intestinal metaplasia (IM) is a recognized high-risk factor for gastric cancer (Watabe et al., 2005; Naylor et al., 2006; Rugge et al., 2010; Sugano et al., 2015). In Correa’s cascade reaction phase, *H. pylori* dominance was the main finding for the microbiota, with a decreasing trend in alpha diversity and gastric microbiota interactions that remained relatively stable after onset of GC (Wang et al., 2020; Yu et al., 2020). Furthermore, the relative abundance of *H. pylori* during chronic gastritis correlated negatively with other phyla, such as Proteobacteria, Firmicutes and Bacteroidetes.

Microarray G3PhyloChip™ analysis of changes in the gastric mucosal microbiota from NAG to IM to GC patients revealed significant microbiota segregation between NAG and GC, with 44 taxa showing changes in abundance (Aviles-Jimenez et al., 2014). Decreases in Porphyromonas, Neisseria, TM7 group, and *S. sinensis*, as well as increases in *L. coleohominis* and Lachnospiraceae, were observed and may have contributed to the development of gastric cancer. A study revealed changes in gastrointestinal microbial diversity and interactions at various stages of gastric precancerous lesions, particularly at the GIN stage (Liu et al., 2021), and network analysis showed that the intensity of intergeneric symbiotic interactions increased in IM and decreased in GIN with gastritis progression. It was also found that the interaction is stronger in IM than in SG (Coker et al., 2018). Moreover, a higher degree of centrality and strong cooccurrence interactions among the genera *Gemella*, *Veillonella*, *Streptococcus*, *Actinobacillus*, and *Haemophilus* were observed in gastric biopsies of gastric precancerous lesions. These results suggest that bacteria tend to interact with each other simultaneously to form specific microecological networks prior to gastric carcinogenesis.

In a recent study, the changes in the microbiota of GM and GF at different disease stages were evaluated in 180 patients with superficial gastritis, intestinal chemosis (IM), and GC (He et al., 2022). The Shannon index and observed species were lower in GC patients than in SG patients in the GF sample. In contrast, the Shannon index of GC was higher than that of SG in the GM samples, with no significant differences observed between the different stages. Further examination of gastric acid at different disease stages revealed elevated pH in GC compared to SG, suggesting that neutralization of the gastric microenvironment promotes diversity in the mucosal microbiota. Microscopic interactions between GM and GF in GC were significant compared to SG, with some genera in GF showing significant positive correlations with their counterparts in GM, including *Helicobacter*, *Streptococcus*, and *Haemophilus*. These results suggest that *H. pylori* plays a crucial role in development of Correa’s cascade and hypogastric acidity, which creates a favorable environment for growth of other microbiota, promoting progression of gastric cancer. Correa’s cascade responds to different stages of gastric microbiota composition, and targeting specific gastric microbiota and manipulating their composition may be a new strategy for GC prevention and treatment.

In addition, the research on the gastric microbiome faces challenges due to a lack of standardized methods for sample collection, processing, and analysis. Different sequencing platforms, sample types, and analysis methods can all affect the comparability of results. Additionally, the choice of 16S rRNA gene sequencing region can impact result accuracy. To mitigate these differences, several measures can be taken. Firstly, standardized sample processing methods should be established to ensure consistent procedures are followed during sample collection and handling. Secondly, a uniform selection of reliable sequencing platforms and analysis methods should be implemented, allowing for similar technical approaches across different studies. Simultaneously, it is important to choose the same region of the 16S rRNA gene for analysis during the sequencing process to ensure result consistency. Moreover, conducting large-scale and multicenter studies that encompass diverse geographical regions and population samples can reduce the impact of geographic and population factors. Encouraging data sharing and promoting collaborative research efforts can facilitate result validation and reproducibility. Finally, the development of consistent standards and guidelines to regulate the methods and reporting in gastric microbiome research will enhance the comparability of research findings.

### 4.2. Gastric cancer

*Helicobacter pylori* is the strongest known risk factor for intestinal and diffuse GC. However, little is known about the sequence of events in diffuse gastric cancer (DGC). Only a few studies have reported that *H. pylori* and/or EBV infection play an important role in development of sporadic diffuse gastric cancer (Uemura et al., 2001; Kwak et al., 2014). In one study, patients with current *H. pylori* infection were more likely to develop DGC than those with previous infection (Misra et al., 2007). Serological studies have shown that patients with high *H. pylori* IgG titers are at greater risk of developing DGC than intestinal gastric adenocarcinoma (Tatemichi et al., 2009; Lee et al., 2017). Several studies have indicated that as *H. pylori* decreases during the gastric cancer stage, there is a corresponding increase in non-*H. pylori* microbiota that promote cancer (Ferreira et al., 2018; Hsieh et al., 2018). This indicates that the effect of *H. pylori* should be fully considered when studying these GC-associated microbiota. The greatest benefit was obtained by eradicating *H. pylori* in patients before development of gastric mucosal atrophy and IM (Ford et al., 2014; Chen et al., 2018). In contrast, no significant benefit was obtained by eradicating *H. pylori* in patients with malignant progression of gastric cancer after IM (Wong et al., 2004; Chen et al., 2016; Malfertheiner et al., 2017; Mera et al., 2018). Hence, cancer may occur even after *H. pylori* eradication therapy (de Vries et al., 2009).

Loss of GC tissue-specific glands and reduced acid secretion lead to depletion of *H. pylori* and significant enrichment of intestinal commensal bacteria, including *Lactobacillus*, *Enterococci*, *Carnobacterium*, *Parvimonas*, *Citrobacter*, *Clostridium*, *Achromobacter*, and *Rhodococcus* (Amieva and Peek, 2016; Hsieh et al., 2018; Gantuya et al., 2020). Interestingly, certain strains of lactic acid bacteria used as probiotics have a complicated interplay with *H. pylori*, inhibiting its adherence to epithelial cells, bacteriocin or organic acid production, and releasing associated inflammatory factors (Kabir et al., 1997; Yang and Sheu, 2012; Sakarya and Gunay, 2014). Nevertheless, some lactobacilli can contribute to the development of gastric cancer. LAB have been shown to stimulate production of exogenous lactic acid, reactive oxygen species (ROS), and N-nitroso compounds, which cause DNA damage and accelerate carcinogenesis, and to increase expression of proto-oncogenes, induce angiogenesis, inhibit the apoptotic process, and enhance the epithelial-mesenchymal transition (EMT), immune tolerance, and colonization by other carcinogenic pathogens (Vinasco et al., 2019; Whiteside et al., 2021). Regardless, there is still a dearth of research that explains the specific mechanisms by which these symbiotic microorganisms promote gastric carcinogenesis.

Several studies have shown an association between specific microorganisms and gastric cancer. Enrichment of the microbial flora from oral or intestinal origin has frequently been observed in the gastric microbiota of GC patients (Hsieh et al., 2018; Hu et al., 2018; Castaño-Rodríguez et al., 2017; Yu et al., 2017). Oral microbiota such as *Fusobacterium*, *Veillonella*, *Peptostreptococcus*, *Streptococcus*, *Slackia*, *Parvimonas*, and *Haemophilus* have been identified in patients diagnosed with gastric cancer (Hu et al., 2018; Chen et al., 2019; Wang et al., 2020). Different combinations of oral microorganisms can be used to differentiate the stages of Correa’s cascade (Coker et al., 2018; Zhang et al., 2021). Liu et al. (2019) investigated the diversity, composition, and bacterial symbiotic correlates and predicted functional profiles of the gastric microbiota in three microenvironments, including 230 normal, 247 perineural, and 229 tumor tissues. They reported that *P. melaninogenica, S. anginosus*, and *P. acnes* are enriched in tumor microhabitats but that *P. copri* and *B. uniformis* are significantly reduced, with *B. fragilis* and *A. muciniphila* showing similar patterns of variation between peritumor and tumor tissues. Most notably, bacterial abundance was found to be reduced in peritumor and tumor microhabitats, and the network associated with enrichment of gastric bacteria in tumor microhabitats was simplified. Moreover, we compared differences in the gastric microbiota between three microhabitats in patients with intestinal, diffuse and mixed gastric cancer. Within the same gastric microhabitat, several nondominant bacterial phylotypes differed between intestinal and diffuse GC types, though the composition of the overall gastric microbiota did not differ significantly. This suggests that the gastric microhabitat of GC, rather than its stage, type or cellular differentiation, determines the overall structure of the gastric microbiota.

Recent clinical studies have also revealed dysregulation of the fungal flora between a tumorigenic gastric mucosa group and normal gastric mucosa in GC (Zhang et al., 2022), with a lower abundance of Pezizomycetes, Sordariales, Chaetomiaceae, and Rozellomycota in the GC group than in the normal group in terms of taxonomic classification. Solicoccozyma was found to be more abundant and differentially enriched. Additionally, a significant increase in *Candida albicans*, which promotes gastric cancer development by decreasing the diversity and abundance of microorganisms in the stomach, in GC was reported (Zhong et al., 2021). These two studies reveal altered GC-associated fungal composition and ecology and demonstrated that *Candida albicans* and Solicoccozyma may be used as fungal biomarkers for GC.

### 4.3. Mechanisms of gastric microbiota in gastric carcinogenesis

Microorganisms in the precancerous state interact with each other through complex mechanisms. The microbial community within the affected tissue undergoes dynamic changes, with certain microorganisms promoting the growth and survival of others. For example, pathogenic bacteria can create an environment conducive to the growth of other harmful microorganisms by producing virulence factors and altering the local pH levels. This alteration in the microbial composition and diversity can lead to dysbiosis, where the balance between beneficial and harmful microorganisms is disrupted. Dysbiosis, in turn, triggers inflammatory responses and compromises the integrity of the host tissue. Additionally, the interplay between microorganisms can result in the production of harmful metabolites, such as genotoxic compounds or reactive oxygen species, which further contribute to tissue damage and genomic instability. The cumulative effect of these adverse mechanisms can drive the progression of the precancerous state toward malignancy.

From current preclinical studies, gastric microbiota has been shown to induce mechanisms such as DNA damage, inflammation, and immune suppression, thereby promoting the development of gastric cancer. In some studies, *Propionibacterium acnes*, associated with acne, has been found to induce DNA damage and inflammatory reactions to promote the development of GC. Propionibacterium acnes and microbial metabolites such as short-chain fatty acids can induce the expression of the NKG2D ligand. These bacteria activate the NKG2D system, leading to a significant upregulation of the pro-inflammatory IL-15, and resulting in autoimmune lymphocytic gastritis (LyG) (Montalban-Arques et al., 2016). *Prevotella* can promote various inflammatory reactions, including GC, by enhancing resistance to host-derived reactive oxygen species and producing redox proteins (Hofer, 2014; Irfan et al., 2020). Additionally, some studies suggest that nitrate-reducing bacteria may contribute to the development of gastric cancer by increasing the concentration of carcinogenic N-nitroso compounds in the stomach. Several bacteria, including *Clostridium*, *Haemophilus*, *Staphylococcus*, *Neisseria*, *Lactobacillus*, and *Nitrospirae*, have been implicated in promoting gastric cancer through the stimulation of N-nitroso compounds (NOC) production (Kaźmierczak-Siedlecka et al., 2022). Moreover, the dysbiosis of the microbiota can modulate the components of the tumor microenvironment. In the GC microenvironment, relevant analyses have indicated that an abundance of Stenotrophomonas and Selenomonas are positively correlated with plasmacytoid dendritic cells (pDC) and regulatory T cells (Tregs), respectively. On the other hand, the abundance of Comamonas and Gaiella is negatively correlated with pDC and Tregs, respectively (Ling et al., 2019).

Research on the role of fungal and viral microbiota in tumors is relatively limited compared to bacteria (Papon et al., 2021). However, studies have identified a significant elevation of *Candida albicans* in GC patients (Zhong et al., 2021). *Candida albicans* may contribute to carcinogenesis through multiple mechanisms, particularly by triggering inflammation and inducing Th17 response. The activation of NF-κB and Wnt pathways, facilitated by IL-17, can create a pro-inflammatory environment that promotes tumor development (Ramirez-Garcia et al., 2016; Dai et al., 2019). EBV can inhibit the proliferation of CD8+ T cells and reduce the cytotoxicity of NK cells, thereby contributing to the development of both acute and chronic gastritis and increasing the risk of tumor formation (Polakovicova et al., 2018; Nie and Yuan, 2020). Additionally, specific EBV miRNAs have been identified that can impact the proliferation of infected cells, raising the risk of malignant tumor formation. For instance, one study discovered that Epstein–Barr virus miR-BART17-5p directly downregulates KLF2, thereby promoting migration and growth of gastric cancer cells (Kim et al., 2015).

These findings highlight the potential involvement of alterations in the gastric microbiota in regulating multiple mechanisms that drive the occurrence of gastric cancer. Nevertheless, further research is necessary to fully understand the intricate mechanisms through which these microbial changes influence tumor development.

## 5. Potential clinical value of the gastric microbiota for gastric cancer diagnosis

Microbiota-based biomarkers for gastric cancer screening, diagnosis, and therapy can be developed through identification of specific gastric microorganisms that are enriched or depleted in GC patients. Currently, studies focusing on gastric microbial models to predict the performance of PLGC or GC have largely centered on *H. pylori*-positive patients. However, few studies have reported significantly altered non-*H. pylori* species for use as potential microbial biomarkers for GC (Coker et al., 2018; Wang et al., 2020; Kadeerhan et al., 2021). Analysis of the microbial characteristics associated with gastric cancer have revealed enrichment of *Proteobacteria*, *Citrobacter*, *Lactobacillus*, *Clostridium*, and *Rhodococcus* in gastric cancer (Ferreira et al., 2018).

Ferreira et al. integrated enriched and deficient taxa in GC as the Microbial Dysbiosis Index (MDI), a gastric microbiota-based diagnostic measure, to differentiate patients with GC from those with chronic gastritis. The superior sensitivity and specificity of MDI for detecting gastric cancer compared to using a single taxonomic unit suggests that changes in the microbial community rather than individual taxa contribute to gastric cancer development. In receiver operating characteristic (ROC) analysis, MDI performed well in identifying gastric cancer, with the highest area under the curve (AUC) of 0.91 for the gastric cancer cohort. Another study constructed a random forest classifier model between GC and SG to assess the diagnostic value of gastric mucosa (GM) and gastric fluid (GF) microbial markers for GC (He et al., 2022). The model was validated using seven GMs (*Lactobacillus*, *Gemella*, *Enterococcus*, *Helicobacter*, etc.) genera and 13 GF genera (*Lactobacillus*, *Filifactor*, *Staphylococcus*, *Dialister*, etc.) as the best marker set. The probability of disease (POD) index was found to be significantly higher for GC than for SG. In ROC analysis, the gastric microbial markers had AUCs of 83–94%. In the validation cohort (including 60 SG and 60 GC patients), the AUCs of the GM markers and GF markers were 84 and 89%, respectively, revealing that the gastric microbial-based classifier was able to accurately differentiate between GC and SG.

To determine whether specific bacterial signatures may be used as a diagnostic tool for gastric cancer, one study analyzed gastric epithelium-associated bacterial species in patients with gastritis, IM and GC and found a sensitivity of 73% and specificity of 100% when using a combination of five species. In contrast, the combination of *C. colicanis, F. canifelinum*, and *F. nucleatum* showed 100% sensitivity and approximately 70% specificity. These data indicate that *C. colicanis* and *F. nucleatum* are enriched in cancer specimens and might identify gastric cancer with 100% sensitivity. This study validated that *C. colicanis* and *F. nucleatum* represent diagnostic markers for detection of gastric cancer (Hsieh et al., 2018).

Although gastric microbes can be used as potential diagnostic markers for GC, use of gastric microbe prediction as an alternative to traditional screening or diagnostic methods for gastric cancer seems remote. Indeed, gastric microbes are influenced by multiple factors, and the accuracy of predictive models as potential microbial biomarkers for GC requires further evaluation and validation in subsequent experimental studies in a broader population to examine whether these specific bacteria contribute to progression of GC.

## 6. Microbiome-based therapeutic approaches for gastric cancer

### 6.1. Probiotics

In the contemporary medical landscape, probiotics and their metabolites have become widely employed for treating human conditions linked to dysbiosis of the gastrointestinal microbiota. Probiotics are defined as live microbial agents that coexist symbiotically within the human host, and when ingested in sufficient amounts, they can have beneficial effects on the host (Hill et al., 2014; Cunningham et al., 2021). Probiotic supplementation is an emerging therapy for *H. pylori* eradication (Francavilla et al., 2014; Goderska et al., 2018; Ji and Yang, 2020). Probiotics comprising the genera Lactobacillus and Bifidobacterium have been shown to reduce adverse effects (e.g., nausea, vomiting, diarrhea, abdominal pain) caused by antibiotic therapy and may improve eradication efficiency while supporting balance in the intestinal microbiota (Javanmard et al., 2018). Over the past decade, immune checkpoint inhibitors such as PD-1/PD-L1, CTLA-4, and LAG-3 have radically transformed management of advanced cancer. The microbiota is intricately involved in regulating the host immune system via diverse signaling pathways, thereby influencing the body’s response to cancer immunotherapy (Ma et al., 2019). In preclinical studies, specific microorganisms have been found to contribute to immunotherapy in conjunction with immune checkpoint inhibitors. In animal models, controlling tumor growth with Bacteroidales alone is comparable to treatment with PD-L1 monoclonal antibody therapy (Weng et al., 2019). Bacteroidales can also boost CTLA-4 blockers to enhance the efficacy of cancer immunotherapy (Vétizou et al., 2015). Several probiotic strains, including *Lactobacillus acidophilus, Lactobacillus bulgaricus*, and *Lactobacillus salivarius*, have been shown to downregulate IL-8 levels, which correspondingly reduces expression of the bacterial tumor protein CagA (Zhou et al., 2008; Yang et al., 2012). Furthermore, probiotics can be utilized to mitigate the side effects of perioperative enteral nutrition in patients with gastric cancer. Clinical trials have established that enteral nutrition supplemented with probiotics not only decreases the incidence of postoperative diarrhea in gastric cancer patients but also augments the immune response (Zhao et al., 2017; Xie et al., 2018). Probiotics can interact with dendritic cells (DCs), activating them and promoting favorable immune responses while suppressing Th1, Th2, and Th17-mediated inflammatory responses. In enterocytes, secretion of TNF-α inhibitory metabolites and blocking NF-κB signaling can reduce production of TLR, leading to a further reduction in the inflammatory response (Raheem et al., 2021). Research has found that *Lactobacillus acidophilus* and *Bifidobacterium longum* can exhibit anti-gastric cancer proliferation and anti-angiogenesis effects by downregulating the expression of COX2 (Nada et al., 2020). In a clinical trial, probiotics can be used as a probiotic supplement in combination with antibiotics and proton pump inhibitors to assist in eradicating *Helicobacter pylori* infection. The results show that the eradication rate of *Helicobacter pylori* in the combination therapy group (88.5%) is significantly higher than that in the monotherapy group (63.3%) (Rafiei et al., 2021).

### 6.2. Fecal microbiota transplantation

Fecal microbiota transplantation (FMT) is transplantation of beneficial flora from the feces of healthy individuals into patients via enema, oral, gastric tube or capsule to restore or improve the patient’s gastrointestinal microecology. FMT has been shown to be effective in treatment of refractory *C. difficile* infections, inflammatory bowel disease and antibiotic-associated diarrhea, as well as other intestinal disorders (Ademe, 2020). To date, numerous studies have identified that FMT modulates the gut microbiome and immune system associated with tumors and may represent a major therapy for advanced cancers (Frankel et al., 2017; Chandra and McAllister, 2021). FMT has yielded encouraging results in animal models and clinical trials. A comprehensive analysis was conducted on the gut microbiota of gastrointestinal (GI) cancer patients (19 colorectal cancer, 23 gastric cancer, 14 esophageal cancer, and 18 other GI cancer types) undergoing PD-1/PD-L1 therapy (Peng et al., 2020). The cohort included 45 responders and 29 non-responders. Regardless of their clinical response, the gut microbiota in the cohort was primarily composed of the phyla *Bacteroidetes* and Firmicutes. In patients with favorable outcomes, there was a higher proportion of *Prevotella*/*Bacteroides*, and the responder subgroup exhibited a higher abundance of *Prevotella*. Research by Routy et al. (2018) established that an abnormal gut microbiota composition is a primary cause of resistance to ICIs. In patients with advanced cancer, administration of oral antibiotics during immunotherapy inhibited the clinical benefit of ICIs. Transplanting fecal microbiota from ICI-responsive cancer patients into germ-free or antibiotic-treated mice improved the antitumor effects of PD-1 blockade (Routy et al., 2018). Currently, a clinical trial (NCT04130763) is underway to investigate the use of FMT to enhance the efficacy of PD-1 therapy in gastrointestinal cancer patients.

### 6.3. Gastric microbiota transplantation

Similar to FMT, it is postulated that gastric microbiota transplantation (GMT) has potential benefits in treatment of cancer. Nevertheless, research on GMT is still in its infancy and presents multiple challenges, as described below.

#### 6.3.1. Microbiota complexity

The gastric environment is characterized by an acidic and hypoxic setting, accompanied by robust gastric acid secretion and peristaltic motility. The composition and abundance of the gastric microbiota are influenced by various factors, including dietary patterns, lifestyle habits, and medication usage. These factors may impede the survival and colonization of transplanted microbial communities, thereby increasing the challenges associated with establishing a stable microbial population within the stomach. Moreover, unlike the intestinal microbiota, the gastric microbiota exhibits lower diversity and abundance, making it challenging to precisely regulate and restructure the microbial community. Introducing the donor microbiota into the recipient’s gastric environment necessitates ensuring the survival of microorganisms under adverse conditions such as gastric acid, while adapting to the ecological milieu within the stomach. The viability and colonization capacity of the gastric microbiota pose significant challenges.

#### 6.3.2. High technical difficulty

The transplantation techniques associated with the gastric microbial community are comparatively intricate, requiring a high level of technical expertise and precise execution. In contrast, fecal transplantation can be prepared through simple procedures such as centrifugation and filtration, with relatively accessible sample sources. However, the acquisition of gastric microbiota necessitates invasive procedures such as endoscopic biopsy or other specialized collection methods, which are intrusive operations involving patient fasting and sedation or anesthesia. During the process of preparing gastric samples, stringent quality control is imperative to ensure the purity, activity, and stability of the microbiota, thereby rendering the attainment of microbiota that meets the required standards a challenging endeavor. The transplantation modality may encompass the introduction of the donor microbiota into the recipient’s stomach through means like oral capsules, nasogastric tubes, or gastroscopy. Different transplantation methods may influence the survival rate and colonization efficacy of microorganisms. Additionally, determining the appropriate dosage of microbiota for transplantation presents a complex issue that necessitates consideration of the gastric capacity for accommodation and the survival rate of the microbiota.

#### 6.3.3. Lack of standardized operating procedures

The research on GMT remains in its nascent stages, lacking standardized operational procedures and therapeutic protocols. Optimal practices concerning the sourcing and quality control of transplant material, transplantation dosage, frequency, and routes are yet to be definitively established. Currently, there is a dearth of standardized methods for preserving microbial samples to ensure their viability and efficacy during transplantation. The preservation and freezing processes of microorganisms require precise control of temperature, oxygen levels, and other environmental factors to prevent microbial inactivation or damage. Furthermore, the lack of standardization has led to variations in transplantation methods among different medical institutions and practitioners, compromising the comparability and reproducibility of outcomes.

#### 6.3.4. Safety

Due to the transplantation of diverse microorganisms involved in GMT, potential safety concerns such as infection, allergic reactions, and rejection responses may arise. Additionally, GMT may give rise to adverse reactions and side effects. These reactions can include allergic responses, gastrointestinal discomfort, and digestive disturbances. Further research is needed to evaluate and understand the specific occurrence rates and severity of adverse reactions and side effects. Currently, limited knowledge exists regarding the long-term safety of GMT. Long-term follow-up and monitoring studies are crucial for assessing the long-term safety of GMT, enabling the identification and resolution of any potential long-term safety issues.

#### 6.3.5. Ethical

GMT is still in its preliminary research stage, and ensuring that the experimental process aligns with ethical principles and safeguards the rights and safety of participants is a crucial challenge. Additionally, acquiring transplant material involves obtaining gastric microbiota samples from donors. When selecting donors, careful consideration must be given to their health status, screening for infectious diseases, and ethical considerations. GMT may potentially benefit the health of recipients, but it also entails inherent risks and uncertainties. Ethical considerations require a balance between the benefits and risks of transplantation, ensuring that the process is based on scientific data and clinical practice.

#### 6.3.6. Cost

Gastric microbiota transplantation technology is still in the research stage, and the cost of treatment is high. More economic investment and research are needed to reduce the cost of treatment and increase the penetration rate.

#### 6.3.7. Evaluation of treatment outcomes

Currently, there is a lack of standardized evaluation criteria and methods to assess the therapeutic efficacy of GMT. Microbiome analysis can provide information about the composition of microbial communities, but further research is needed to determine how to interpret this data and relate it to treatment outcomes. Additionally, quantifying improvements in patient symptoms, gastric mucosal histopathological changes, and other relevant indicators poses a challenge. There are significant variations in the composition and functionality of microbiota among individuals. Individual factors, including genotype, lifestyle, and dietary habits, contribute to potential variations in the therapeutic effects of GMT among different patients. Therefore, predicting the long-term effects of GMT and the duration of microbial community stability post-transplantation remain challenging.

Recently, only one study on GMT has been reported by a Korean researcher. This study entailed transplantation of the gastric microbiota from individuals with diverse gastric states into germ-free (GF) mice, whereby it was discovered that the gastric microbiota from individuals with intestinal chemosis or gastric cancer (GC) selectively colonized the mouse stomach and induced precancerous lesions (Kwon et al., 2022). Conversely, if healthy human gastric microbiota is transplanted into the stomachs of mice with distinct gastric diseases, what alterations would be made to the disease status of the mice? This is an issue that requires investigation. To understand this issue, we are conducting a clinical study (ChiCTR2200066339) on the effect of GMT for eradicating *H. pylori* infection. Probiotics and FMT/GMT represent current areas of focus in the field of microecological therapy, with these microbial transplantation techniques projected to serve as effective therapies for gastric cancer and other diseases in the future. To enhance the success of probiotic or FMT/GMT therapy, it is vital for researchers to gain a comprehensive understanding of the systemic impact of the microbiota on the immune system, as well as the relationship of the microbiota with cancer treatment.

## 7. Conclusion

Gastric cancer is one of the most common cancers in the world, and the mortality rate remains high. The discovery of gastric microbiota characteristics in various clinical conditions, from normal stomach to precancerous lesions and GC development, may significantly impact our understanding of the carcinogenic process of GC. The presence or absence of specific microbial communities is closely associated with the occurrence and progression of GC. The existence of these microorganisms may impact gastric cancer development through various mechanisms, including chronic inflammation, immune modulation, and metabolic changes. Therefore, by targeting the dysbiosis of the gastric microbiota, we may be able to develop novel treatment approaches, such as microbiota transplantation, probiotics, and antibiotics, to reduce the risk of GC or slow down disease progression. Although some progress has been made in studying the human gastric microbiota in recent years, the causal relationship between the gastric microbiota and gastric cancer has not been fully established, and the research in this field is still limited. Future investigations should focus on delving deeper into the mechanisms underlying the dysbiosis of the gastric microecology and its role in gastric cancer development. Thus, there is an urgent need for large-scale, multicenter, prospective studies to elucidate the dynamic changes of the microbiota within the Correa’s cascade and identify specific microorganisms that could serve as potential biomarkers for GC and alternative indicators for monitoring disease progression. This provides new avenues for early screening and diagnosis of GC, while also offering guidance for determining optimal treatment strategies and timing.

In conclusion, the gastric microbiota holds significant potential in the diagnosis and treatment of GC. Through in-depth research on the changes in the gastric microbiota and its association with GC, we can provide new insights and approaches for early screening, diagnosis, and treatment of GC. This will contribute to improving patient outcomes and providing personalized and precise management strategies for GC.

## Author contributions

HH, WZ, XW, YY, TW, RC, and YL drafted the preliminary manuscript. FH and JL refined and approved the final manuscript. All authors contributed to the article and approved the submitted version.

## Funding

This research was supported by the Sichuan Provincial Department of Science and Technology, under Grant number 22NSFCS1601. The funding organization had no role in the design of the study, data collection, analysis, or in the writing of the manuscript.

## Conflict of interest

The authors declare that the research was conducted in the absence of any commercial or financial relationships that could be construed as a potential conflict of interest.

## Publisher’s note

All claims expressed in this article are solely those of the authors and do not necessarily represent those of their affiliated organizations, or those of the publisher, the editors and the reviewers. Any product that may be evaluated in this article, or claim that may be made by its manufacturer, is not guaranteed or endorsed by the publisher.
